# Stimulation of Myc transactivation by the TATA binding protein in promoter-reporter assays

**DOI:** 10.1186/1471-2091-6-7

**Published:** 2005-05-05

**Authors:** John F Barrett, Linda A Lee, Chi V Dang

**Affiliations:** 1 Department of Medicine, Johns Hopkins University School of Medicine, Baltimore, Maryland 21205, USA; 2 Department of Cell Biology, Johns Hopkins University School of Medicine, Baltimore, Maryland 21205, USA; 3 Department of Pathology, Johns Hopkins University School of Medicine, Baltimore, Maryland 21205, USA; 4 The Sidney Kimmel Comprehensive Cancer Center, Johns Hopkins University School of Medicine, Baltimore, Maryland 21205, USA

## Abstract

**Background:**

The c-Myc oncogenic transcription factor heterodimerizes with Max, binds specific DNA sites and regulates transcription. The role of Myc in transcriptional activation involves its binding to TRRAP and histone acetylases; however, Myc's ability to activate transcription in transient transfection assays is remarkably weak (2 to 5 fold) when compared to other transcription factors. Since a deletion Myc mutant D106-143 and a substitution mutant W135E that weakly binds TRRAP are still fully active in transient transfection reporter assays and the TATA binding protein (TBP) has been reported to directly bind Myc, we sought to determine the effect of TBP on Myc transactivation.

**Results:**

We report here a potent stimulation of Myc transactivation by TBP, allowing up to 35-fold transactivation of reporter constructs. Although promoters with an initiator (InR) element briskly responded to Myc transactivation, the presence of an InR significantly diminished the response to increasing amounts of TBP. We surmise from these findings that promoters containing both TATA and InR elements may control Myc responsive genes that require brisk increased expression within a narrow window of Myc levels, independent of TBP. In contrast, promoters driven by the TATA element only, may also respond to modulation of TBP activity or levels.

**Conclusion:**

Our observations not only demonstrate that TBP is limiting for Myc transactivation in transient transfection experiments, but they also suggest that the inclusion of TBP in Myc transactivation assays may further improve the characterization of c-Myc target genes.

## Background

The c-*myc *oncogene is implicated in the genesis of many human cancers and accounts for about 70,000 US cancer deaths annually [1-3]. This oncogene produces the c-Myc transcription factor, which heterodimerizes with Max via the helix-loop-helix-leucine zipper (HLH-Zip) motif to bind specific target DNA sequences and regulate transcription [4-8]. The amino-terminal region of c-Myc, when tethered to the yeast GAL4 DNA binding domain, behaves as a potent transactivation domain (TAD) [9]. On the other hand, the transactivation potential of native c-Myc appears diminished when compared with other transcription factors, such as the HLH-Zip protein USF1 or to the GAL4 chimeric transactivators [10].

The basis for the diminished transactivation potential of c-Myc has remained elusive despite the discoveries that the Myc activation domain specifically binds to factors such as TRRAP [7,8,11-13]. TRRAP is a high molecular weight, multifaceted molecule that is capable of recruiting the histone acetyltransferase GCN5 [12]. The fact that c-Myc is able to transactivate in *Saccharomyces cerevisiae *and that yeast Tra1 is similar to TRRAP suggest that Myc's ability to transactivate in yeast may involve Tra1 [14,15]. The c-Myc TAD encompasses two conserved regions, termed Myc Box I and Myc Box II. Although Myc Box I does not appear to affect transactivation, Myc Box II is required for interaction with TRRAP. Although deletion of Myc Box II renders Myc defective in binding TRRAP, it does not affect the ability of Myc to transactivate specific promoter-reporter constructs and in particular it does not affect the ability of GAL4-Myc chimeric protein to transactivate [9]. In addition, deletion of Myc Box II appears to affect the induction of certain endogenous target genes but not others [16,17]. These observations suggest that transcriptional regulation by Myc is likely to be manifold, involving chromatin modulation as well as direct interaction with components of the basal transcriptional machinery [18-20]. This spectrum of activities allows Myc to regulate subsets of genes that are more tightly controlled and susceptible to chromatin modulation, whereas other genes, such as the so-called "housekeeping" genes, may already exist in open chromatin configuration and hence may be regulated through recruitment of the basal transcriptional machinery.

Searches for the interaction of c-Myc with components of the transcriptional machinery have uncovered an interaction with the coactivator CBP [21]. The C-terminal region of Myc has been found to interact with SWI/SNF5 and Miz-1, both implicated in transactivation and transrepression activities of Myc [22-27]. However, these activities could not account for the transactivation potential of the Myc N-terminal region (TAD). The interaction between Myc and the TATA binding protein (TBP) has been observed in diverse systems with evidence from intracellular chemical crosslinking, mammalian two-hybrid assay, yeast two-hybrid assay, and GST fusion protein pull-down assays [28-33]. In fact, two recent studies suggest that the Myc TAD is consisted of a structureless N-terminal (Myc_1-88_) portion connected by a linker followed by a C-terminal (Myc_92-167_) partly helical domain, such that the two domains are induced to adopt a specific conformation upon binding TBP [31,32]. On the basis of these observations, we sought to determine in this study whether TBP is limiting for Myc transactivation in transient transfection experiments.

We sought to characterize the functional interaction of the Myc TAD with TBP using the chimeric GAL4-Myc fusion proteins as well as full-length Myc with four model promoters (adenoviral major late promoter (AdML), lactate dehydrogenase A (LDHA), CDK4 and ornithine decarboxylase (ODC)) [34-37]. We found that addition of a TBP expression vector in the transactivation assays increases the transactivation by c-Myc from several fold to well over 30-fold [38]. By contrast, a GAL4-USF1 transactivator did not respond to increasing input TBP. We also observe different responses by promoters that contain initiator (InR) sequences versus promoters that only contain TATA elements [39-41]. Furthermore, a Myc Box II point mutation W135E does not affect the ability of GAL4-Myc fusions to synergize with TBP [42], but the deletion mutant D106-143 has a blunted effect with cotransfected TBP. Our findings not only support a functional interaction between c-Myc and TBP, but they also provide a means to improve transient transfection assays to study c-Myc target genes.

## Results

### TBP is limiting for GAL4-Myc transactivation

**Figure 1:**
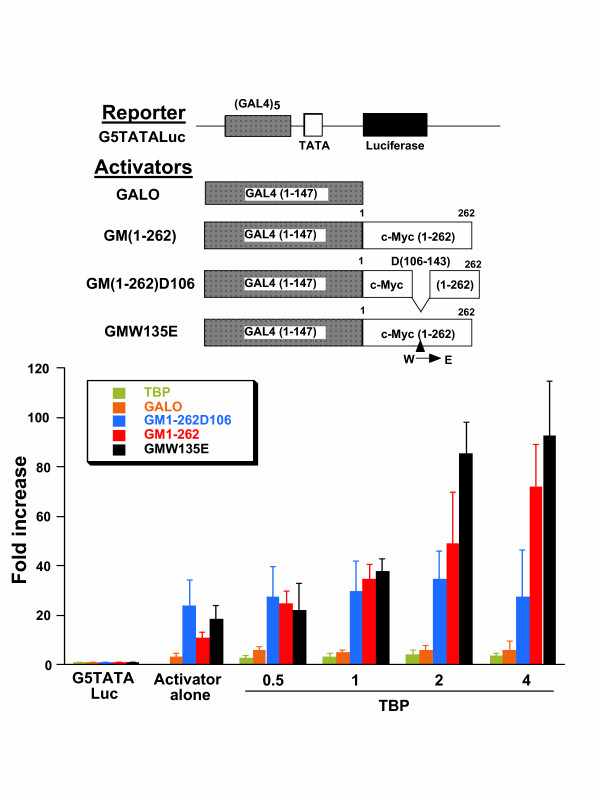
TBP stimulates GAL4-Myc transactivation. The reporter G5TATALuc contains five GAL4 binding sites followed by a minimal TATA box and the luciferase cDNA. Activator alone: the reporter was cotransfected with GAL4(1-147) DNA binding domain (GALO), GAL4 fused to Myc residues 1-262 (GM1-262), GM1-262 with residues 106–143 deleted (GM1-262D106), or GM1-262 with a substitution of tryptophan 135 to glutamate (GMW135E) alone. TBP at increasing input plasmid amounts (μg indicated on the abscissa) was cotransfected into CHO cells with the reporter alone (TBP) or with the indicated GAL4 plasmids. Bars are shown as averages with standard deviation (n = 10).

To determine whether TBP is limiting, we titrated the GAL4-Myc transactivation assays with increasing amounts of TBP plasmid. Although the GAL4-Myc chimera has significant transactivation activity under the experimental conditions chosen, addition of increasing amounts of TBP plasmid resulted in a corresponding increase in transactivation of GAL4-luciferase reporter (G5TATALuc) from about 10 fold to over 60 fold with the highest amount of TBP plasmid used (Fig. 1). TBP itself does not affect the basal activity of G5TATALuc reporter, indicating that the effect of TBP requires the presence of the GAL4-Myc chimera. Further, the GAL4 DNA binding domain (GALO) with minimal transactivation activity was not affected by increasing TBP.

Since Myc Box II is necessary for interaction with TRRAP, we sought to determine whether mutations in this region affect the response of the Myc TAD to TBP. While the deletion D106-143, which removes critical residues of Myc BoxII, activates the reporter better than wild-type Myc TAD, this deletion renders Myc TAD non-responsive to increasing input TBP (Fig. 1). A substitution mutation in Myc Box II, W135E, which was previously shown to have diminished interaction with TRRAP and diminished transformation activity [42,43], has a robust response to increasing TBP. These results suggest that the entire region comprising residues 106–143 is required for synergy with TBP, whereas the transformation defective W135E mutant still responds to increasing input TBP.

### GAL4-Myc synergy with TBP is dependent on the TATA element

**Figure 2:**
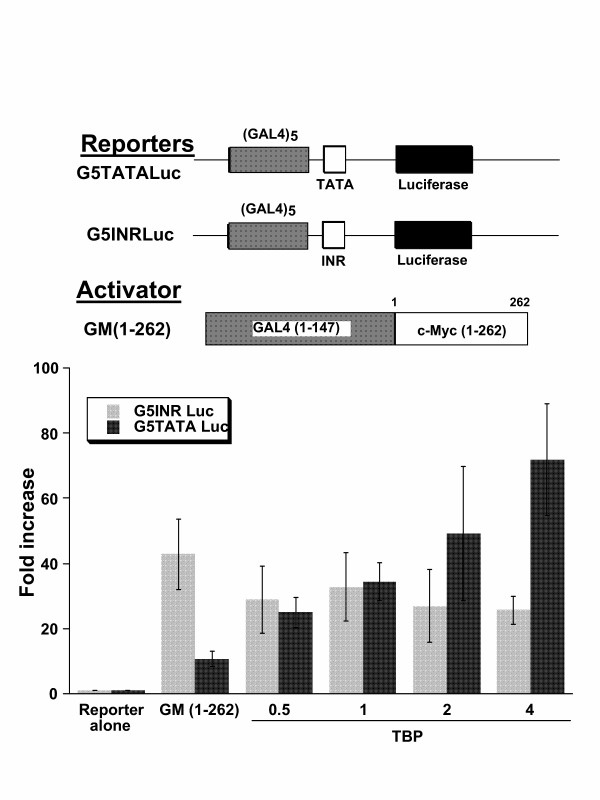
TBP does not stimulate initiator driven luciferase reporter G5INRLuc that contains five GAL4 binding sites. Either G5INRLuc or G5TATALuc (see Fig. 1) was cotransfected into CHO cells with GM1-262 alone or with increasing amounts of TBP (μg indicated on the abscissa). For comparison, data points for G5TATALuc are the same as those shown in Fig. 1. Note that TBP does not stimulate GAL4Myc transactivation of G5INRLuc; there is a slight inhibition by TBP. Bars are shown as averages with standard deviation (n = 4).

Since TBP was shown to be limiting for TATA-dependent but not InR-dependent transcription [44], we sought to determine whether GAL4-Myc cooperation with TBP requires the TATA element. We compared the response of a GAL4 dependent reporter that is driven by an InR element (G5INRLuc) with one that is driven by a TATA element (G5TATALuc) (Fig. 2). In contrast to the TATA driven reporter, the InR-driven reporter responded more briskly to GAL4-Myc but the reporter activity did not further increase with TBP. This observation suggests that the Myc TAD activates InR dependent transcription, for which TBP is not limiting.

### TBP is not limiting for USF1 TAD

**Figure 3:**
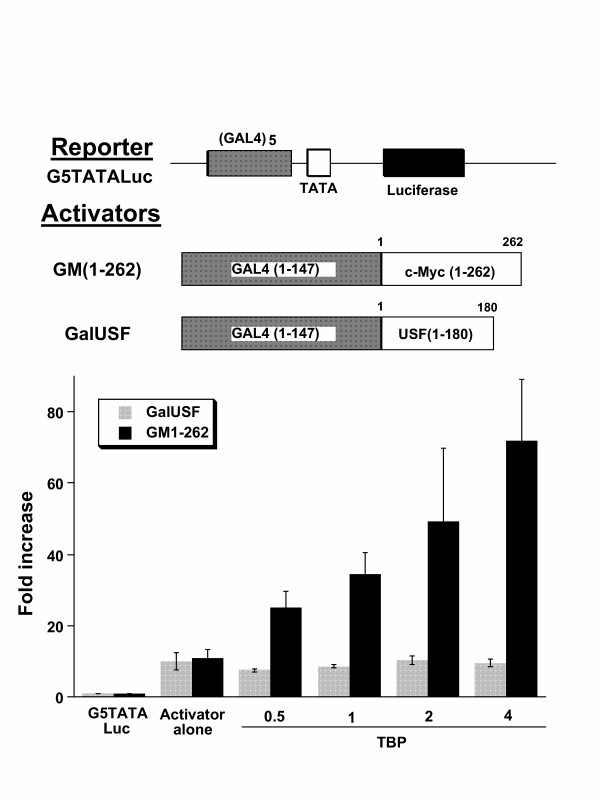
TBP does not stimulate the activation of G5TATALuc by GAL4USF1. For comparison, the stimulation of G5TATALuc by GAL4Myc (GM1-262) and TBP is shown. By contrast increasing amounts of TBP (μg indicated on the abscissa) does not increase reporter activity that is stimulated by USF TAD. Bars are shown as averages with standard deviation (n = 4). Plasmids were transfected into CHO cells.

To determine whether the effect of TBP is selective, we compared the ability of the USF1 TAD with the Myc TAD to synergize with TBP. USF1 is a transcription factor that also belongs to the HLH-Zip family and either homodimerizes or heterodimerizes with USF2. The USF family members binds to DNA target sites (5'-CACGTG-3') that appear indistinguishable from Myc targets, though Myc is capable of binding additional non-canonical sites. To eliminate the effects of the USF1 and Myc DNA binding domains on transcription, we sought to study chimeras of GAL4 with USF1 or Myc TADs. Although the GAL4-USF1 chimera activates G5-TATA-Luc as effectively as GAL4-Myc, TBP did not further increase the activity of GAL4-USF1 (Fig. 3). This observation suggests that while USF1 and Myc may have overlapping target DNA binding sequences, their TADs appear distinctly different, particularly in response to the addition of TBP.

### Effects of TBP on full-length wild-type and mutant c-Myc transactivation of the LDHA promoter

**Figure 4:**
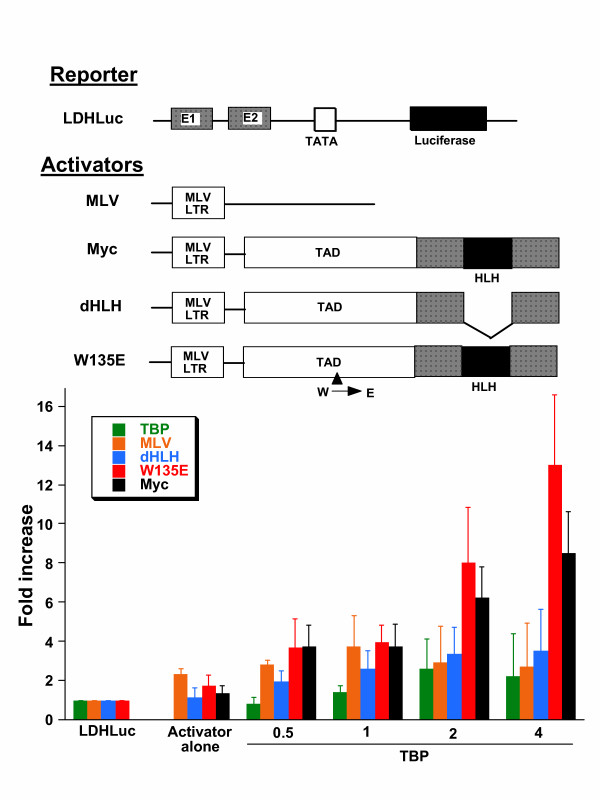
Activation of the lactate dehydrogenase A promoter construct (LDHLuc) by Myc is increased by TBP. Activator alone: cotransfection with LDHLuc and MLV-LTR driven Myc expression vectors: MLV, empty expression vector; Myc, wild-type Myc; dHLH, helix-loop-helix deletion mutant D371-412; W135E, substitution mutant with glutamate replacing tryptophan 135. Increasing amounts of TBP (μg indicated on the abscissa) was cotransfected with Myc and reporter constructs. Bars are shown as averages with standard deviation (n = 10). Transfections were performed using NIH 3T3 cells.

Having observed a TATA-dependent synergy between TBP and the Myc TAD in the chimeric GAL4 system, we sought to determine whether TBP could stimulate Myc transactivation of target gene promoters. We first chose the LDHA promoter that comprises two canonical E-boxes located about 100 and 200 bp upstream of the transcriptional start site [36]. We used an amount of input MLV-LTR-driven Myc expression vector that only minimally increased LDHA promoter activity to study the effects on increasing input TBP. TBP increased wild-type Myc activity from only about 20 % to about 800 % increase (Fig. 4). TBP alone, without added Myc, slightly increased reporter activity to about 2-fold. Note that the empty MLV LTR plasmid caused a slight increase in reporter activity, which was not accentuated by the addition of TBP.

We also studied two Myc mutants in the context of additional TBP (Fig. 4). The Myc dHLH mutant lacks the helix-loop-helix domain, and therefore neither dimerizes with Max nor binds DNA. The dHLH mutant minimally affects basal promoter activity and was not affected by increasing input TBP. The W135E mutant contains a substitution in the Myc Box II domain that renders Myc deficient in transformation [42,43]. Intriguingly, W135E was active and fully responsive to increasing TBP.

**Figure 5:**
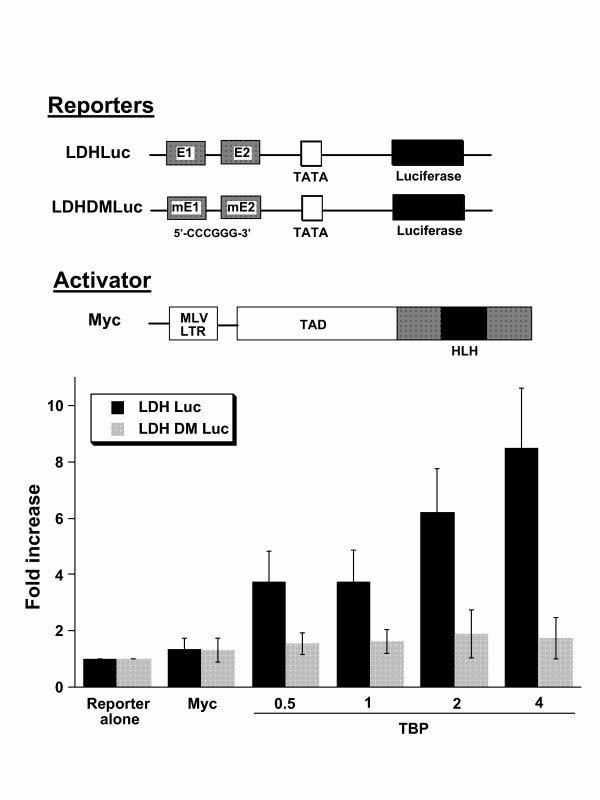
Myc DNA binding sites in the LDHA promoter construct (LDHLuc) is required for TBP stimulation of Myc-mediated transactivation. Wild-type LDHLuc or a mutant promoter construct (LDHDMLuc), which has both Myc E-boxes mutated from 5'-CACGTG-3' to 5'-CCCGGG-3', were cotransfected with a constant amount of MLV-LTR driven wild-type c-*myc *plasmid and increasing amounts of TBP (μg indicated on the abscissa). Bars are shown as averages with standard deviation (n = 6). Transfections were performed using NIH 3T3 cells.

Because transient transfection reporter assays are confounded by many factors, we sought to assure that the synergistic transactivation of the LDHA promoter by Myc and TBP is dependent on Myc binding. We compared the response of the wild-type LDHA promoter (LDH Luc) with one in which both E-boxes are mutated (LDH DM Luc) so that Myc could not bind these sites (Fig. 5). The mutant LDH DM Luc displayed no increase in reporter activity with increasing amounts of TBP in contrast to the wild-type LDH Luc. This result confirms that the synergy between TBP and Myc is dependent on the Myc binding sites in the LDHA promoter.

### The effect of the initiator (InR) element on the synergy between Myc and TBP

We have previously studied the adenoviral major late (AdML) promoter as a model Myc responsive promoter that contains both InR and TATA elements [37]. Others and we have shown that c-Myc regulation of the AdML is biphasic with transactivation followed by transrepression at high levels of input Myc plasmids [37,45]. The transactivation phase depends on E-boxes, whereas the transrepression phase depends on the InR. Here we chose the AdML promoter to determine the effect of the InR on the synergy between Myc and TBP.

**Figure 6:**
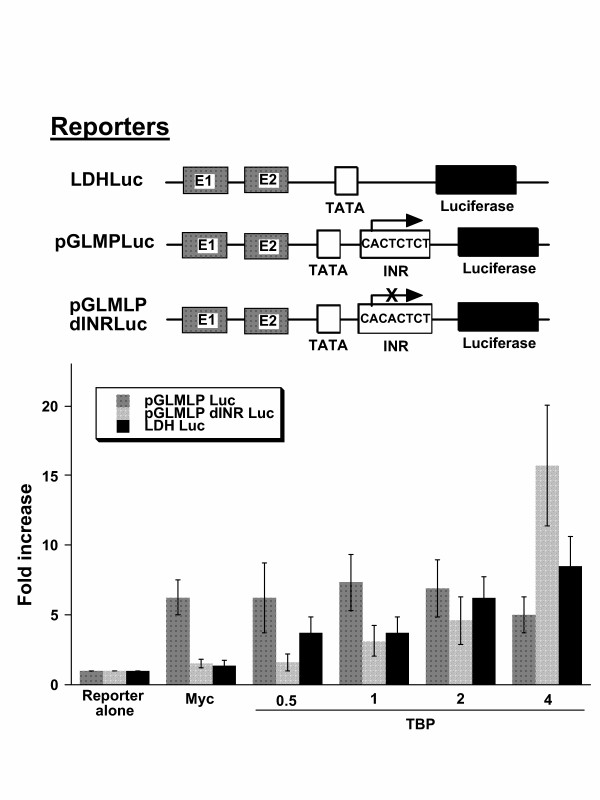
The initiator element in the adenoviral major late promoter luciferase construct (pGLMLP Luc) increases the response to Myc but does not allow further stimulation by TBP. Compared with the reporter containing a mutant InR (pGLMLP dINR Luc), which displays a highly synergistic stimulation by Myc and TBP, the wild-type pGLMLP Luc construct briskly responds to Myc independent of increasing amounts of TBP (μg indicated on the abscissa). Bars are shown as averages with standard deviation (n = 10). Transfections were performed using NIH 3T3 cells.

As compared with the LDHA promoter, which increased in activity with increasing input TBP, the AdML promoter briskly increased activity in response to Myc alone but did not further increase with TBP (Fig. 6). Mutation of the InR element in AdML promoter renders it much less responsive to Myc, but addition of TBP resulted in a marked increase in promoter activity. These observations suggest that the InR increases promoter response to Myc alone, but the InR renders the promoter independent of increasing TBP. Hence, it may be surmised that the combination of E-boxes and InR may be optimal for promoters that require sharp response to Myc regulation, since TBP would not be limiting.

### Synergy of Myc and TBP with CDK4 and ODC responsive sequences

**Figure 7:**
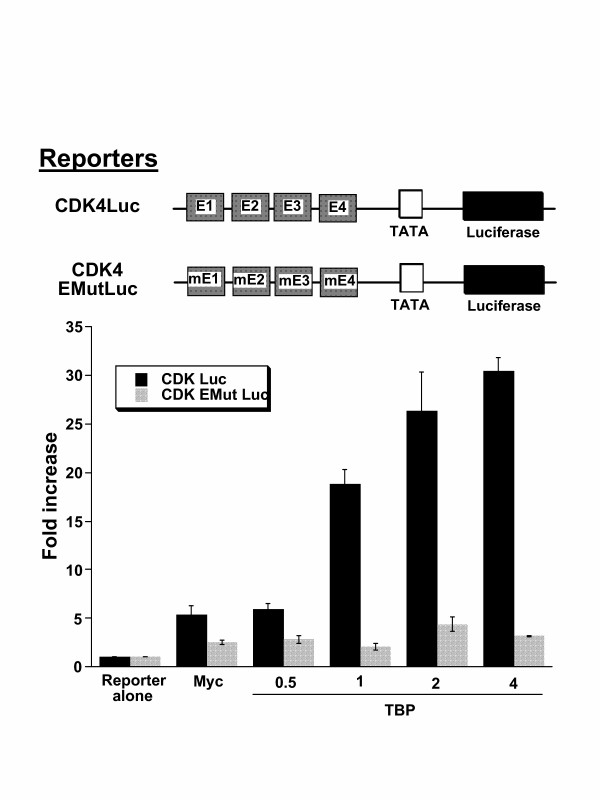
Activation of the human CDK4 promoter (CDK Luc) by Myc is further stimulated by increasing amounts of TBP (μg indicated on the abscissa). The synergy between Myc and TBP is dependent on the Myc binding sites, which are mutated in the reporter CDK EMut Luc. Bars are shown as averages with standard deviation (n = 6). Transfections were performed using NIH 3T3 cells.

We selected the CDK4 promoter, which contains four canonical E-boxes and no InR element, and the intronic ODC tandem E-box sequence to determine the extent of synergy between Myc and TBP. The CDK4 promoter responded to Myc as previously reported. Increasing input TBP further caused a 30-fold activation of the CDK4 promoter as compared to 5-fold activation with Myc alone (Fig. 7). The synergy between Myc and TBP is dependent on Myc binding, since the E box mutant CDK4 promoter is not responsive to the combination of Myc and TBP.

**Figure 8:**
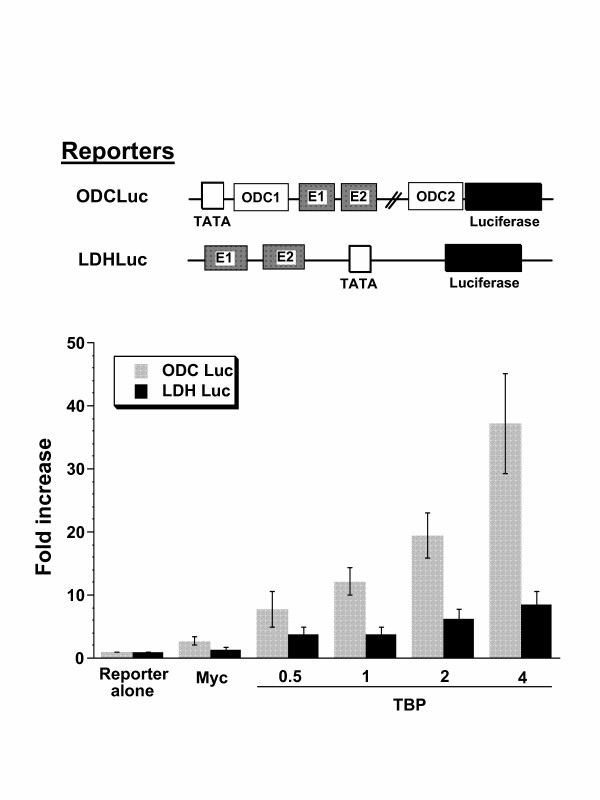
Activation of the ornithine decarboxylase (ODC) intronic E-boxes driven luciferase reporter (ODC Luc) by Myc is further stimulated by TBP (μg indicated on the abscissa). The relative marked stimulation of the ODC sequences by Myc and TBP is compared with the more diminished stimulation of the LDHA promoter (LDH Luc) previously shown in Fig. 4. Bars are shown as averages with standard deviation (n = 10). Transfections were performed using NIH 3T3 cells.

**Figure 9:**
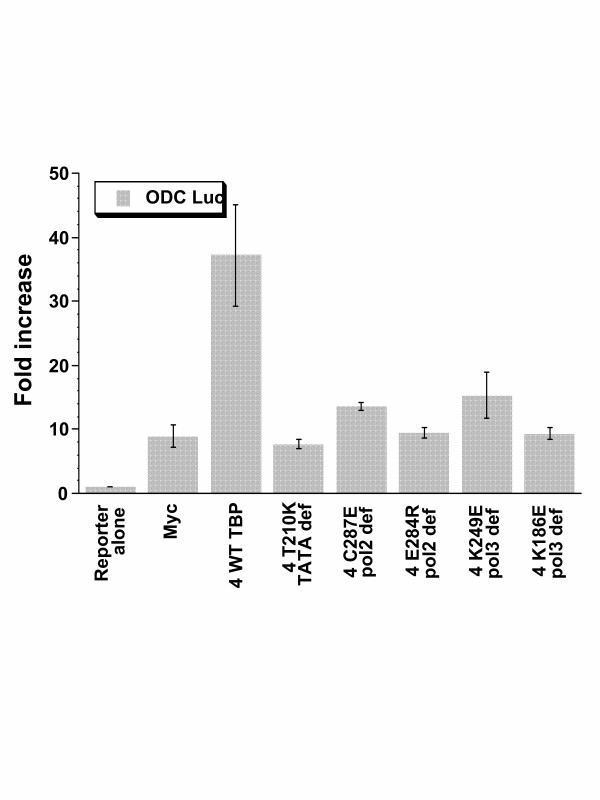
Synergy between Myc and TBP to activate ODC Luc (see Fig. 8) is diminished by mutations in TBP that inhibits TATA box binding (TATA def), interaction with RNA polymerase II (pol2 def) or interaction with RNA polymerase III (pol3 def). Myc (1 μg) was cotransfected with ODC Luc and 4 μg of wild-type or mutant TBP constructs. Bars are shown as averages with standard deviation (n = 4). Transfections were performed using NIH 3T3 cells.

ODC, the prototypical Myc responsive gene, provides a different model that utilizes intronic Myc binding sites. The ODCLuc reporter comprises the ODC promoter and intronic E boxes with flanking sequences. Compared to the LDHA promoter, which displayed about an 8 fold induction, ODCLuc responded to TBP and Myc with a 35-fold induction (Fig. 8). With this robust response, we sought to determine the response of ODCLuc to Myc and TBP mutants (Fig. 9). In this experiment, ODCLuc was co-transfected with a constant amount of Myc and the maximal amount of input TBP. Whereas wild-type TBP stimulated ODCLuc, all TBP mutants studied had virtually no activity (Fig. 9). The lack of activity of TBP mutants defective in TATA binding or Pol II interactions was expected; however, we are intrigued by the lack of apparent activity of the Pol III defective TBP mutants. Further studies will be necessary to define the molecular basis for the lack of synergy between Myc and these various TBP mutants. We conclude however, that wild-type TBP is necessary for synergy with Myc in the transactivation of ODC Luc.

## Discussion

Myc's dramatic biological effects, a plethora of well-characterized interactions between Myc and other proteins, an ever-expanding list of putative target genes and a seemingly weak transactivation potential characterize the enigma of c-Myc-mediated gene regulation [4,46]. Compared with other more potent transactivators, especially in the same family of HLH-Zip proteins, c-Myc stimulates reporter constructs only 2- to 5-fold in an E-box dependent manner. The basis for this apparently weak transactivation is poorly understood. We report in this paper a strong synergy between Myc and TBP resulting in up to 35-fold induction of reporter plasmids. Our observations indicate that TBP is limiting for Myc transactivation and provide a means to enhance the characterization of Myc target genes.

The weak transactivation potential of c-Myc may well be biologically significant when the degree of gene induction by c-Myc is considered [8]. The emergence of an increasing number of Myc target genes reveals several characteristics among the genes. With only a few exceptions, Myc induces endogenous genes by only a few fold above background. In multiple instances, it appears that the broad-based effect of inducing multiple genes in the same pathway by c-Myc may be more important than the marked induction of a few genes [7,47]. Perhaps c-Myc globally affects gene expression through multiple mechanisms. The connection between c-Myc and histone acetylation has become more firmly established, suggesting a role for Myc to modulate chromatin [47-51]. Beyond chromatin modulation, the role of Myc in transcriptional initiation or elongation is less well understood. Searches for an interaction between Myc and members of the general transcription factors have revealed an interaction between the Myc transactivation domain and TBP [31]. In the work reported here, we provide evidence for a functional interaction between Myc and TBP in transient transfection reporter assays. Although the addition of TBP dramatically enhances these assays, the biological significance of this synergism is not delineated in our study. In particular, since many Myc target genes are induced only several fold *in vivo*, the role of TBP in modulating these target genes *in vivo *is not at all clear.

In response to TBP and Myc, promoters with an initiator element respond differently compared with those with a TATA element only [39-41]. With both the GAL4 chimeric proteins and full length Myc, InR driven promoters respond to the Myc TAD briskly. However, the increase in TBP did not further augment the activities of InR driven promoters. These observations are consistent with previous findings that TBP is limiting for TATA driven, but not InR driven promoters in *Drosophila *[44]. It is intriguing to note the initial brisk response of InR containing promoters to Myc, which at high levels can inhibit the same InR driven promoters. We surmise from these findings that promoters comprising both TATA and InR elements may control Myc responsive genes that require brisk increased expression within a narrow window of Myc levels independent of TBP. Such genes would be sharply induced by Myc, which in excess can inhibit the same genes through the InR [37,45].

In contrast to InR containing promoters, promoters with TATA element only, such as CDK4 and LDHA, increase in activity with increasing TBP levels in the presence of a constant amount of Myc. These promoters may be regulated by the activity of TBP *in vivo*, although evidence for this is lacking. The observation that oncogenic Ras can augment TBP activity suggests that a subset of Myc target genes may also be further responsive to increased TBP through activated oncogenic Ras [52]. In fact, Myc and Ras can cooperate to regulated cdc2 [53]. Hence, it will be instructive to determine the set of Myc responsive genes versus the set of genes that are responsive to both Myc and Ras. Comparison of promoters or regulatory regions of these genes are likely to uncover a level of transcriptional complexity previously unappreciated.

The fact that the synergy between TBP and Myc was observed with the GAL4 chimeric activator system and full length Myc suggests that the synergy is mediated through the Myc transactivation domain. Furthermore, the Myc Box II deletion mutant D106-143 was unresponsive to increasing TBP, indicating that this region of Myc is required for synergy with TBP. This observation is consistent with the previous finding that in vitro interaction between Myc and TBP requires Myc Box II [28]. Although we observed a significant synergy between Myc and TBP, none of the TBP mutants retained any synergistic activity. It is not surprising that both TATA box binding mutant and Pol II interaction defective TBP mutants were dysfunctional. Although it may seem surprising that Pol III interaction defective TBP mutants were also inactive, recent studies suggest a significant overlap between Pol II and Pol III interactions with TBP [38]. Although beyond the scope of the current study, it will be of significant interest to map the regions of TBP required for the interaction with Myc and correlate this with the ability for TBP mutants to synergize with Myc.

## Conclusion

In summary, we describe in this report a significant stimulation of Myc transactivation by TBP. However, the presence of an InR diminishes the promoter response to TBP. We surmise that these differences may be exploited *in vivo *to increase the complexity and range of gene regulation by Myc.

## Methods

### Plasmids

GAL4 constructs were as described [9]. GAL4-MycW135E (GMW135E) is GAL4(1-262) in which the Pst1-Pst1 fragment was exchanged with a fragment containing the substitution W135E from full length c-*myc *in MLVMycW135E [43]. GAL4-USF1 was constructed by inserting a PCR-amplified, sequence-verified 560-bp USF1 fragment (corresponding to residues 1–180) into the NdeI-BstEII sites of pGALm. The USF1 cDNA template for PCR was a gift from M. Sawadogo and R. Roeder [10]. The Gal4 TATA-driven reporter G5TATALuc was constructed from G5TATA-CAT(gift from M. Green) by replacing CAT with luciferase (Luc) [54]. The Gal4 InR-driven G5INRLuc reporter (gift from J. Gralla) was as described [55].

Murine sarcoma virus long terminal repeat (MSV-LTR) promoter driven wild-type and mutant TBP expression vectors were gifts from A. Berk and are as described [56]. Expression vectors for wild-type and mutant c-*myc *are as described [43].

The reporter ornithine decarboxylase ODC-Luc is a gift from J. Cleveland [35]. The wild-type and mutant lactate dehydrogenase promoter LDH-Luc reporters were previously described [36]. Wild-type and mutant adenoviral major late promoter AdML-Luc constructs were previously reported [37]. Wild-type and mutant cyclin dependent kinase CDK4-Luc was described [34].

### Cell culture and transfection

Chinese Hamster Ovary (CHO) cells were grown in 5% CO2 at 37°C in αMEM

(LTI) supplemented with 10% fetal bovine serum (LTI) and antibiotics as described [9]. Cells were transiently transfected using DEAE-dextran (0.275 mg/ml). Two μg of GAL4 reporter luciferase plasmid, two μg of GAL4 chimeric activator plasmid and increasing amounts of pLTRTBP (0.5 to 4 μg) were cotransfected into 100 mm plates of 60% confluent CHO cells. DNA concentration was maintained at 8 μg by the addition of pLTR empty vector. Cells were incubated with the DNA in serum-free DEAE-dextran/MEM media overnight. Cells were harvested 48 hours after DMSO/ chloroquine shock and assayed for luciferase activity as described.

NIH3T3 cells (gift from R. Eisenman) were grown in 5% CO2 at 37°C in DMEM (low glucose) (LTI) supplemented with 10% fetal bovine serum and antibiotics. Cells were transfected with Lipofectin (LTI). Lipofectin was added at 5 X the total concentration of DNA to serum-free Opti-MEM media (LTI) and incubated at room temperature for 45 min. Two μg of various promoter-reporter luciferase plasmid and one μg of full-length wild-type or mutant c-*myc *activator plasmid were added with increasing amounts of pLTRTBP (0.5 to 4 μg) to the Opti-MEM/Lipofectin mixture. DNA concentrations were maintained at 7 μg by adding pLTR empty vector. The Opti-MEM/ Lipofectin/ DNA mixture was incubated at room temperature for 10 min. then added to 100 mm plates of 30% confluent NIH3T3 cells. Cells were incubated for 5 hours, aspirated and fed fresh media and harvested after 48 hours for luciferase activity.

### Luciferase assay

Luciferase activity was measured using the luciferase assay system according to manufacturer's instructions (Promega). Cells were washed in phosphate buffered saline (PBS), scraped using Cell Lysis Solution (Promega) and centrifuged for 2 min at 1000 rpm. Luciferin cocktail (80 μl) was added to 20 μl lysate and luciferase activity was measured in a luminometer. Samples were run in duplicate.

## Authors' contributions

J. Barrett and L.A. Lee performed experiments. All authors designed experiments, interpreted data, participated in writing the manuscript and approved the final version.
